# Size, spines, and primes: the drivers of collar spine numbers among echinostome trematodes

**DOI:** 10.1017/S0031182025000046

**Published:** 2025-01

**Authors:** Bronwen Presswell, Priscila M. Salloum, Jerusha Bennett, Katherine E. Buschang, Robert Poulin

**Affiliations:** Department of Zoology, University of Otago, Dunedin, New Zealand

**Keywords:** attachment, body size, comparative study, natural selection, phylogenetic conservatism, symmetry

## Abstract

Some anatomical structures vary greatly in number among species, a phenomenon that often remains unexplained. We investigate interspecific variation in the number of collar spines among trematodes from the superfamily Echinostomatoidea, using a dataset comprising hundreds of species. These trematodes possess a ring of spines around their anterior sucker; in some families, they form 2 arcs on either side of the sucker, with a central gap, whereas in other families, they form a continuous collar with no gap. First, we confirm that even numbers of spines are the norm among species in which they are arranged as 2 arcs with a central gap, while odd numbers (mainly prime numbers) predominate among species in which spines form a continuous collar. Second, we tested whether variation among species in the number of spines might reflect selective pressures. The spines serve to attach the worm to the inside lining of the host gut. Our analysis confirms that spine numbers correlate positively with worm body size among echinostomes, supporting the hypothesis that larger worms require more spines for stronger attachment. Finally, we tested whether phylogenetic conservatism may explain interspecific variation in the number of collar spines, i.e. whether closely related species have more similar numbers of spines than expected by chance due to shared ancestry. Our analysis confirms that spine numbers show strong phylogenetic conservatism across species. Overall, our findings indicate that the number of collar spines, a hallmark of echinostomes, is the product of conserved phylogenetic inheritance overlaid by adaptive functional adjustments.

## Graphical abstract

**Figure:**
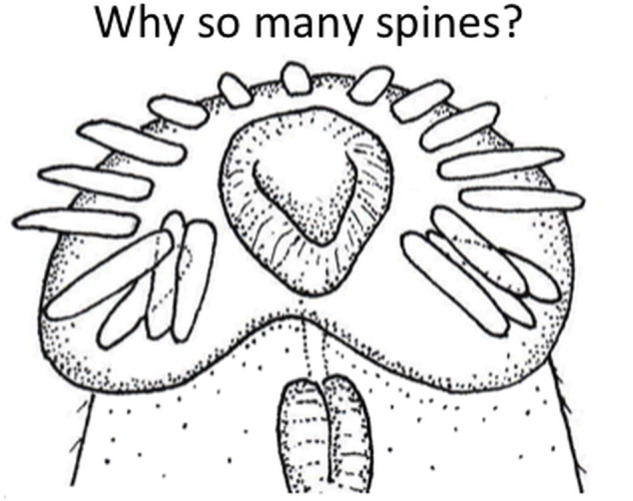

## Introduction

Within any animal taxon, the number and arrangement of major body structures are determined by a general body plan shared across all members of that taxon. For example, sea stars and other echinoderms generally possess 5 (or multiples of 5) arms arranged radially, and arachnids have 4 legs on each side of their bilaterally symmetrical body. These numbers are conserved across species within these higher taxa. Alternatively, the number of particular body structures can vary widely even among related species, in response to species-specific evolutionary pressures or developmental constraints. For instance, in segmented animals such as polychaetes or myriapods (millipedes and centipedes), the number of appendages is a simple function of the number of segments, and thus of body length. Similarly, the number of vertebrae varies extensively among both fish species (Lindsey, 1975) and snake species (Lindell, 1994) in proportion to their body sizes, as does the number and types of teeth among mammalian orders (Line, 2003) in relation to their diet.

Many animals possess secondary structures other than those playing fundamental support or locomotory roles. For these, there are at least 3 processes that can either control the number of structural elements in individual organisms and drive evolutionary divergence among species, or constrain that number to a limited set of values. First, the body plan of a given taxon can determine the possible numbers of structural elements observed among species. For instance, in bilaterally symmetrical animals, we might invariably expect an even number of such structures, arranged in matched pairs on either side of the body. Odd numbers should not be seen in bilaterally symmetrical body plans, unless an additional structure occurs along the central axis of the body. Second, phylogenetic conservatism can limit variation in the number of structural elements among closely related species (Pagel, 1999), even constraining the action of natural selection (McKittrick, 1993). Generally, closely related species share similar trait values due to shared evolutionary history. Accordingly, species within a clade may have all inherited a particular number of structural elements from a common ancestor, with only limited deviation from the ancestral number observed among extant species (e.g. Burroughs, 2019). Third, as with vertebral number in fish and snakes or appendages in segmented animals, we might expect an animal’s body size to select for low or high numbers of particular structural elements. When small, repeated structural elements perform a key functional role, with each additional element improving the combined performance of that function, and when the required performance is related to body size, natural selection may favour a larger number of structural elements in larger species. For example, because heavier birds require greater lift, and thus greater total wing surface area, in order to achieve flight, the number of feathers generally correlates positively with bird size (Hutt and Ball, 1938; Møller, 2015). Feathers are not simply larger in larger birds, they are more numerous. Similarly, acanthocephalan parasites live in the gastrointestinal tract of their vertebrate host, inserting their hooked proboscis into the gut wall to anchor themselves and avoid being dislodged by food passing through the gut. Larger worms are likely more susceptible to being dislodged and therefore require stronger attachment to the gut wall. Presumably as a result of selection, there is indeed a strong positive correlation among acanthocephalan species between worm body size and both the number of sclerotized hooks on their proboscis and the total cumulative length of those hooks (Poulin, 2007), thus resulting in attachment strength proportional to body size.

**Figure 1.**
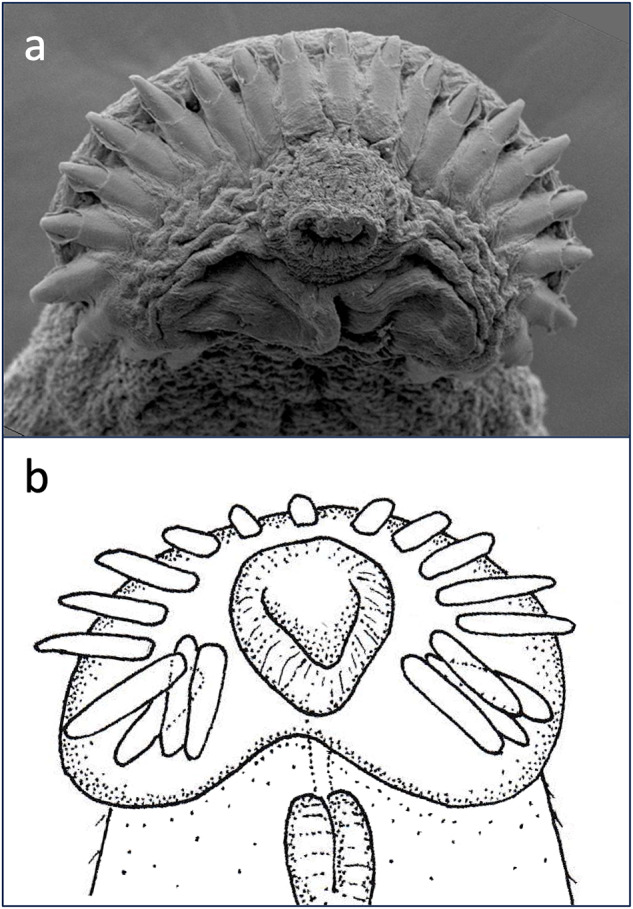
Two examples of collar spines around the oral sucker of echinostome trematodes from New Zealand. (a) Scanning electron micrograph of the anterior end of *Acanthoparyphium* sp. from the South Island pied oystercatcher, *Haematopus finschi*; some spines at both extremities of the collar ring are not clearly visible. (b) Line drawing of the anterior end of *Neopetasiger neocomensis* from the Australasian crested grebe, *Podiceps cristatus.*

Here, we investigate interspecific variation in the number of collar spines among echinostome trematodes (phylum Platyhelminthes, superfamily Echinostomatoidea), considering each of the above 3 explanations. We focus on species within the families Echinostomatidae, Himasthlidae, Caballerotrematidae, Echinochasmidae and Philophthalmidae, in which the presence of collar spines is the norm (Himasthlidae, Caballerotrematidae and Echinochasmidae used to be considered subfamilies of Echinostomatidae; Tkach et al., 2016). These trematodes generally (but not always) use endothermic vertebrates (birds or mammals) as definitive hosts, where they infect the gastrointestinal tract. They are characterized by a ring of spines, or collar spines, arranged symmetrically around their anterior (oral) sucker (see Figure 1). In Echinochasmidae, the spines are arranged as 2 arcs on either side of the sucker, with a gap in the centre of the collar; in the other families, they generally form a continuous collar with no gap, i.e. the gap is filled by 1 or more central spines. These spines consist mostly of crystalline material surrounded by a thin tegument-like layer; muscle bundles at the base of each spine control their protrusion and retraction (Fried et al., 2009). The only recognized function of these spines is to allow the worm to attach securely to the lining of the host gut (Fried et al., 2009). Because their number, sizes and precise arrangement vary greatly among genera (see Kanev et al., 2009), they play an important role in taxonomy and species discrimination (Kostadinova and Gibson, 2000; Kostadinova, 2005).

Based on the 3 processes mentioned above that may either control the number of collar spines and shape evolutionary divergence among species, or constrain that number to a limited range of values, we test 3 predictions across echinostome species: (i) even numbers of collar spines should be the norm in Echinochasmidae, in which a central gap splits the collar into 2 arcs, whereas odd numbers should predominate in the other families, given the worms’ bilateral symmetry with 1 or more extra spines along the central body axis; (ii) numbers of collar spines should correlate positively with worm body sizes, given the presumed role of collar spines in worm attachment; and (iii) spine numbers should be more similar among phylogenetically closely related species than expected by chance alone, assuming significant inheritance of this trait from common ancestors. We tested these predictions by compiling a large dataset on the body size and number of collar spines of echinostome species and combined it with existing phylogenetic information (e.g. Tkach et al., 2016; Chai et al., 2020). In addition to testing the above predictions, we also explored the data for other patterns among numbers of spines, after an examination of the data revealed an unexpected trend (see Results). Overall, our analysis provides the first general explanation of the wide interspecific variation in this hallmark trait of echinostomes.

## Methods

### Data collection

A list of species from the superfamily Echinostomatoidea was obtained from the WoRMS database (https://www.marinespecies.org/), including only ‘accepted’ species names. A few additional species not listed in WoRMS that were found during the subsequent literature search were added to the list. After excluding species which apparently did not have collar spines, based on their description, the final dataset comprised 679 species. While this does not account for all known echinostomes (i.e. the WoRMS database includes mostly marine species) and some of the included species may be invalid, our list nonetheless provides a large and, for our purposes, most likely unbiased sample of echinostome species. These were arranged following the higher taxonomical scheme proposed by Tkach et al. (2016).

Morphometric data on each species were obtained from original species descriptions wherever possible. In some cases where this was not possible, data for a focal species were extracted from tables in other species descriptions where new species were compared with the focal species. When links to original descriptions or redescriptions were not available in WoRMS, we looked for original sources by either searching for species names (and their synonyms) in Google Scholar, or searching the Biodiversity Heritage Library (https://www.biodiversitylibrary.org/) using the ‘advance search’ by ‘scientific names’ function. The following information was recorded for each echinostome species: (i) The family to which it belonged. (ii) The species name of the definitive host and the higher taxon to which it belonged, here simplified to 3 groups: mammal, bird or ectotherm (including fish and reptiles). (iii) The number of collar spines. When a range of values was given, for analysis we used the maximum number, to account for the possibility that some spines had been lost from the specimens on which fewer spines were counted during fixing or slide-mounting. In many cases, a single count was provided, because either a single specimen was measured, no intraspecific variation was observed, or the authors reported only a mean value or a generalized value; in such cases, the single count was used for further analysis. (iv) The body length of adult specimens, again using either the maximum value when a range was given, or the single value provided. (v) The body width of adult specimens at their widest point, using either the maximum value when a range was given, or the single value provided. From these measures, we computed a more appropriate measure of body size: (vi) The body surface area of adult worms (1 side only), which was assumed to approximate a flattened ellipsoid and calculated as (*LW*π)/4, where *L* corresponds to body length and *W* to body width.

Out of the 679 initial entries in our dataset, data were available on spine numbers for 630 species and available for body surface area for 625 species. However, information for 1 or both of these variables was unavailable or incomplete for many species. These species were excluded from the generalized linear model (GLM) described below. Furthermore, we excluded many additional species whose adults had been described based on worms grown in experimental hosts such as domestic chickens, domestic ducks or rats, or in cases clearly identified as accidental human infection; morphometric data from these specimens may not be representative of adults infecting natural hosts. Therefore, the final dataset used in the GLM comprised 513 species.

### Data analysis

All analyses were conducted in the R environment v4.2.2 (R Core Team, 2024). Frequency distributions of worm body sizes (body surface area) and spine numbers were visualized for the complete dataset and also only for trematodes with body surface area smaller than 50 mm^2^ (5·0e^7^ µm^2^), using *ggplot2* (Wickham, 2016). We first tested for general patterns in spine numbers. When differences in the frequency of species with either even or odd numbers of collar spines were clear-cut, no statistical test was necessary. When testing was necessary (i.e. difference in frequency of species with either prime or non-prime numbers of spines), a Chi-squared test was used.

Second, we tested for a relationship between the number of collar spines and worm body size using a GLM with Poisson error structure (for count data) and with a log link function, using the *glm* function implemented in R. Note that because of the irregular distribution of spine numbers, we also ran the GLM using a negative binomial structure; although this yielded a slightly better fit (see Supplementary Table S1), it produced essentially the same results as the one with a Poisson structure, and only the latter is presented below. In the GLMs, the number of collar spines was the response variable, whereas worm body size (surface area) and host taxon (3 levels: mammal, bird or ectotherm) were the predictors. In addition to the main effects model, we conducted a second GLM to check if the interaction between host taxa and body surface area influenced spine number (formula = spines ∼ area*host). The resulting GLM predictions were visualized by using the *ggpredict* function in the R package *ggeffects* (Lüdecke, 2018). Model fit was evaluated based on the deviance and Akaike information criterion (AIC). In addition, a likelihood ratio test was done to compare the 2 models, using the *lrtest* function in the R package *lmtest* (Zeileis and Hothorn, 2002).

Finally, we tested whether spine numbers were more similar among phylogenetically closely related species than expected by chance alone. Indeed, numbers of collar spines may be consistently low or high across species within certain echinostome lineages, via trait inheritance from a common ancestor. We constructed an echinostome phylogeny including as many of the species in our dataset as possible by creating a 28S gene alignment for species within the superfamily Echinostomatoidea. This included species with reported spine numbers that were included in either Tkach et al. (2016) or obtained from NCBI GenBank searches (https://www.ncbi.nlm.nih.gov/genbank/) using each genus name within Echinostomatoidea and either ‘28S’ or ‘large subunit’ keywords. Sequences were aligned in Geneious Prime 2024.0.2 using the Multiple Alignment using Fast Fourier Transform (MAFFT) function (Katoh and Standley, 2013). The alignment was manually trimmed and resulted in 1063 bp in total. The best substitution model of evolution was estimated in the software jModelTest version 2.1.10 (Darriba et al., 2012) and the model GTR + I + G was selected from AIC weights. Bayesian Inference was performed using MrBayes version 3.2.7 (Ronquist et al., 2012) on the online interface CIPRES Science Gateway v.3.3 (Miller et al., 2010). Markov Chain Monte Carlo chains were run for 10,000,000 generations with the first 25% of trees discarded as burning. After the analysis, mixing and convergence estimates were evaluated through stdout files to ensure appropriateness of phylogeny. Family level relationships followed those of Tkach et al. (2016). The resulting phylogeny (.nexus) file was imported into R Studio from a Nexus file via the *ape* package (Paradis and Schliep, 2019) and pruned with the *drop.tip* function to match species in our dataset.

Then, we mapped spine numbers onto the phylogeny and computed the phylogenetic signal (Pagel’s *λ*) in spine numbers across these species. This index serves to assess trait evolution and correlation with evolutionary distance. It ranges from 0, which would indicate no phylogenetic structure in spine numbers, to 1, which would represent strong phylogenetic conservatism and thus closely related species having more similar numbers of spines than expected by chance. Pagel’s *λ* was computed using the *phylosig* function of the *phytools* R package (Revell, 2012). Subsequent visualizations were conducted in R using the *phytools, ggtree* (Yu et al., 2017), *ape*, and *plotrix* (Lemon, 2006) packages and the *contMap* function to produce a color-coded tree showing spine number distribution across species.

## Results

**Figure 2.**
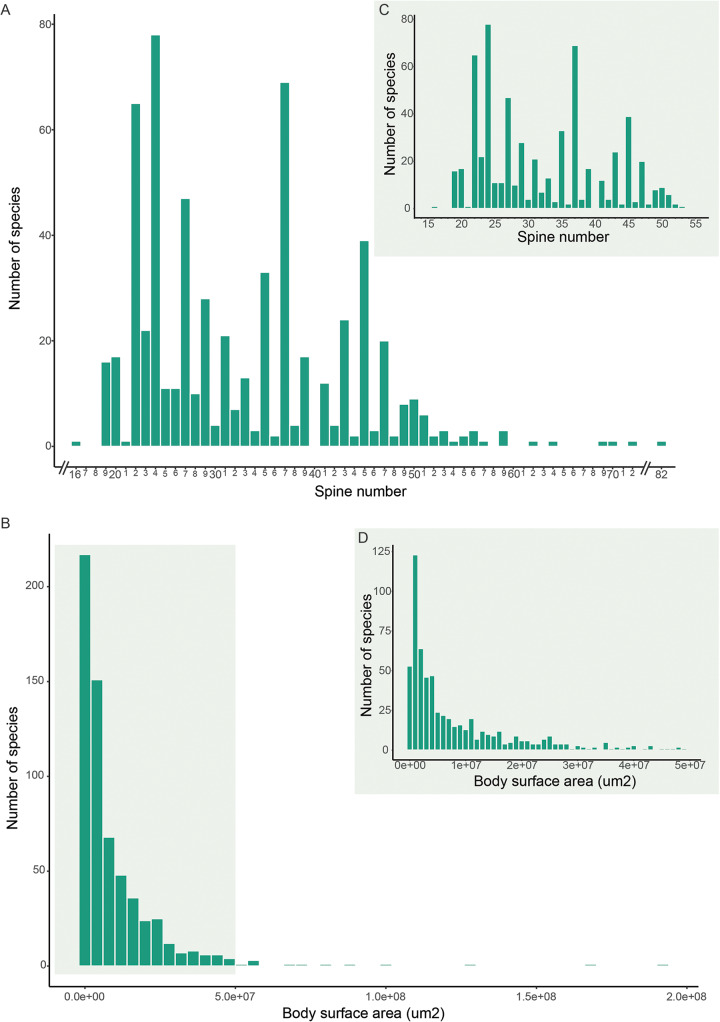
Frequency distributions of spine number and body surface area (µm^2^) among echinostome trematodes. (A) All species with spine number data. Note that the x-axis scale was truncated for visualization purposes where there were large intervals between data points; (B) All species with body surface area data. The shaded area refers to species with body surface area smaller than 5 × 10^7^ µm^2^, which were used to produce panels C and D; (C) Spine number distribution for species with body surface area smaller than 5 × 10^7^ µm^2^; D) Distribution of body surface area for species smaller than 5 × 10^7^ µm^2^.

Among the 630 species in the dataset for which data were available, spine numbers ranged from 16 to 82 (Figure 2A). Among the 513 species used in the GLM for which both spine number and body size (body surface area) data were available, there was a 4000-fold variation in body sizes (Figure 2). The majority, i.e. 432 species (84·3% of total) had bird definitive hosts, with 50 (9·7%) using mammals and 31 (6%) using ectotherm hosts.

There were 152 species of the family Echinochasmidae for which data were available on spine number. As expected for this family, based on the arrangement of collar spines in 2 arcs with a central gap rather than an uninterrupted collar, all except 2 species were reported as having an even number of collar spines. The 2 exceptions may be erroneous counts, or the species were assigned to the wrong genus. Among the 478 species of other families, the majority (395 or 83%) have an odd number of collar spines. The remaining 83 species are reported as having an even spine number; although this may be due to errors in some cases, in some genera (e.g. *Nephrostomum, Ignavia, Ruffetrema*; the latter 2 genera originally assigned to Echinochasmidae and later reclassified) a central gap exists in the collar, and so an even number of spines is expected.

Intriguingly, among the 395 above species with an odd number of collar spines, the reported count of collar spines is a prime number in 217 cases and a non-prime number in 178 cases. The bias towards prime numbers is slightly significant (Chi-squared = 3·85, 1 df, *P* < 0·05), despite the fact that there are more non-prime than prime numbers (14 vs 12) among odd numbers within the range of collar spine values seen in those species (from 19 to 69 spines, inclusively). When adjusting the expected frequencies to reflect the excess of non-prime numbers, the bias towards prime numbers becomes highly significant (Chi-squared = 12·25, 1 df, *P* < 0·001).

**Figure 3.**
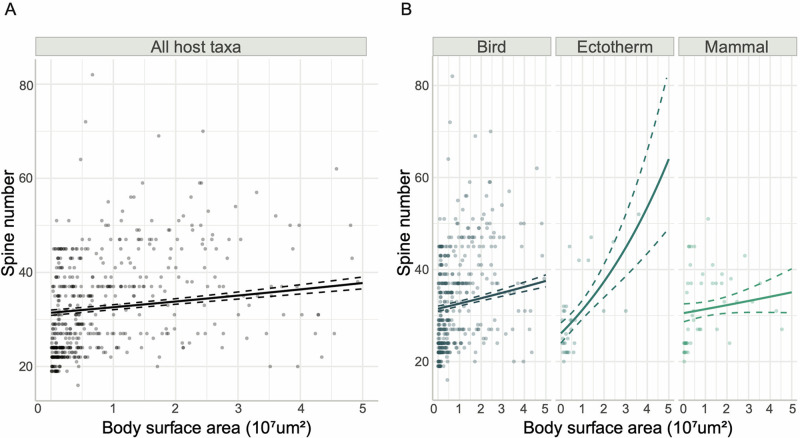
Relationship between the number of collar spines and body size (surface area) among echinostome trematodes, showing the data (points) and predictions (solid lines) from generalized linear models (GLMs) with 95% upper and lower confidence intervals (dotted lines). Note that 9 data points with body surface area larger than 5 × 10^7^ µm^2^ were removed for visualization purposes (all data points were included in the models). (A) Main effects model showing the effect of body surface area on trematode spine number for all host taxa considered together; (B) interaction model showing the interacting effect of body surface area and host taxa on trematode spine number.

The frequency distribution of spine numbers across echinostome species is far from regular but appears Poisson-like, whereas body sizes followed a more regular, highly skewed unimodal distribution indicating a strong bias towards smaller body sizes (Figure 2B and 2D). Notably, the 2 distributions do not resemble each other. The GLM considering the interaction between host taxa and body surface area provides a slightly better (but significant) fit to the data than the main effects model (Supplementary Table S2). The likelihood ratio test supports this result, with a slightly higher loglikelihood (less negative) for the interactions model (Supplementary Table S3). The GLM models suggest that there is a small, but strongly significant, positive effect of body surface area on spine number, therefore trematodes with larger bodies are likely to have more collar spines (Figure 3A, Table 1). In addition, the effect of trematode body surface area on spine number is stronger when the trematode’s host is an ectotherm than when it is a bird (Figure 3B, Table 1). However, when the host is a mammal, trematode body surface is not correlated with spine number (Figure 3B, Table 1).

**Table 1.** Results of the generalized linear models (GLMs) for both the main effects and the interaction models testing the effects of trematode body size (surface area, i.e. area) and host taxon (ectotherm, birds or mammals, with birds as the reference level) on the number of collar spines. Significant effects are in bold

Model	Factor	Estimate	Std. Error	*z* value	*P*-value
Main effects	**Intercept**	**3**·**448e^+00^**	**9**·**241e^−03^**	**373**·**093**	**<2e^−16^**
	**Area**	**3**·**661e^−09^**	**3**·**838e^−10^**	**9**·**538**	**<2e^−16^**
	**Ectotherm**	**−7**·**325e^−02^**	**3**·**385e^−02^**	**−2**·**164**	**0**·**0305**
	Mammal	−3·971e^−02^	2·649e^−02^	−1·499	0·1339
Interaction	**Intercept**	**3**·**449e^+00^**	**9**·**312e^−03^**	**370**·**429**	**<2e^−16^**
	**Area**	**3**·**513e^−09^**	**3**·**999e^−10^**	**8**·**785**	**<2e^−16^**
	**Ectotherm**	**−1**·**863e^−01^**	**4**·**362e^−02^**	**−4**·**272**	**1**·**94e^−05^**
	Mammal	−3·037e^−02^	3·376e^−02^	−0·900	0·368
	Area*Ectotherm	**1**·**441e^−08^**	**3**·**219e^−09^**	**4**·**476**	**7**·**60e^−06^**
	Area*Mammal	−7·566e^−10^	1·748e^−09^	−0·433	0·665

**Figure 4.**
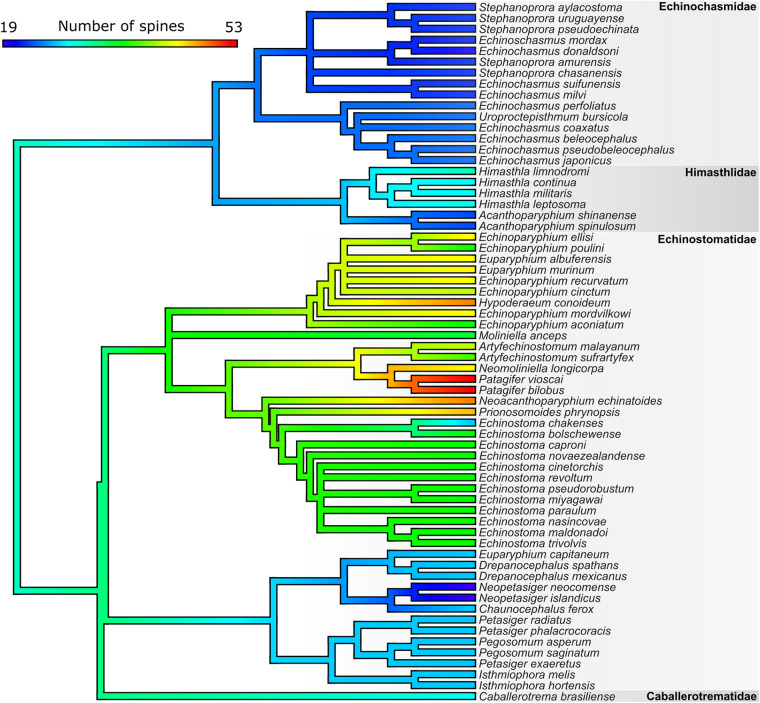
Phylogenetic relationships among echinostome species, showing the distribution of numbers of collar spines across extant species.

Our 28S phylogeny of echinostomes included 64 species (Supplementary Table S4). The distribution of spine numbers among species shows evidence of phylogenetic conservatism, with closely related species having similar if not identical number of spines (Figure 4). This is confirmed by a strong phylogenetic signal (Pagel’s *λ* = 0·977). However, increases in spine numbers have occurred along some branches of the tree (e.g. genera *Himasthla, Patagifer*) whereas decreases have occurred along other branches (genus *Neopetasiger*), suggesting some degree of evolutionary divergence (Figure 4).

## Discussion

Beyond the number and arrangement of major anatomical structures shared by all species within a higher taxon due to a common body plan, there is often substantial interspecific variation in the number and arrangement of secondary structures. In the present study, we investigated the extensive variation in the number of collar spines among echinostome trematodes, focusing on general patterns and some possible underlying processes that may explain them. Specifically, in addition to confirming the connection between the arrangement of collar spines and their number, we also tested whether their number reflected a functional role and/or phylogenetic conservatism.

As we acknowledged earlier, although our dataset includes hundreds of species, it does not include all described species of echinostomes. In addition, some species included in our dataset may be synonyms of each other, leading to a few cases of pseudoreplication. For instance, many currently accepted species in the genus *Echinostoma* have been described from single specimens and lack genetic characterization; they may prove to be invalid species, a common fate among helminth species in general (Poulin and Presswell, 2024). Nevertheless, given the size of the dataset, we feel these issues are unlikely to greatly affect our results.

We found that even numbers of collar spines were observed in the vast majority of species within the family Echinochasmidae, as we expected from the arrangement of their spines in 2 identical arcs separated by a gap along the central axis of the worm’s body. In bilaterally symmetrical animals, structures forming mirror images on the right and left sides of the body arise inevitably from gene regulatory networks as well as proximate tissue-shaping factors during ontogenesis (Holló, 2017). In some animals with multiple secondary structures arranged on either side of their central body axis, the central gap is filled by an additional similar structure, resulting in an odd number in total. For instance, the opossum *Didelphis virginiana* has a centrally positioned extra nipple, resulting in a total of 13 nipples (Stewart et al., 2020). The situation is the same regarding collar spines in echinostomes other than those in the family Echinochasmidae and a few other genera (e.g. *Nephrostomum*). In those other echinostomes, extra spines filling the central gap result in an odd number of spines overall. For instance, in the genus *Echinostoma*, 3 central spines connect the 2 sets on either side of the body, resulting in an uninterrupted collar with an odd number of spines. Reports of species with even numbers are widely considered to be incorrect, the product of missing, retracted or supernumerary spines, or erroneous counts (Kanev et al., 2009).

Strangely, among the nearly 400 species with an odd number of spines, there were significantly more species with a prime number of spines than with a non-prime number. Prime numbers (i.e. any number that is only divisible by 1 and by itself) are distributed seemingly randomly along any continuous series of integers (Savitsky, 2024). They are certainly not a feature of morphology or other biological phenomena. We cannot think of any genetic, evolutionary, developmental or other mechanism or explanation for this finding. The possibility that they might be attractors in morphospace, i.e. trait values along the continuum of possible values towards which species phenotypes converge, can be dismissed. Indeed, why would possessing, say, 37 collar spines provide a trematode with significantly greater fitness, through better attachment strength, than having 35 or 39 spines? Similarly, and for the same reasons, we can rule out the possibility that having a prime number of spines promotes higher diversification rates, such that lineages with prime numbers of spines would proliferate over time and give rise to more species than those with non-prime numbers of spines. We can only surmise that this finding has no particular biological relevance and is instead due to chance.

We tested whether variation among species in the number of collar spines might reflect the action of natural selection, which would be expected to optimize this number based on the selective pressures acting on worms. The presumed function of collar spines is to allow the worm to attach securely to the inside lining of the definitive host gut (Fried et al., 2009). One would expect that larger worms require stronger attachment, and thus more spines. Our analysis confirmed that indeed larger worms tend to have more spines; the overall relationship between body size and spine number is not very strong, as seen from the scatter of points in Figure 3, but highly statistically significant. Various factors can explain the scatter of points, i.e. why some echinostomes have more or fewer spines than expected for their size. For example, some genera like *Parallelotestis* and *Pegosomum* have relatively wide bodies for their length as well as few collar spines, however they live in the gall bladder or bile ducts of their hosts, where they do not face dislodgement by passing food. There may also be variation among species with respect to the length, diameter and/or pointiness of spines, which may for instance allow a worm to achieve efficient attachment with fewer spines than expected for its body size. In addition, collar spines can also vary with respect to their position: there are indeed angle spines, lateral spines and dorsal spines, all pointing in slightly different directions (Kanev et al., 2009). The presence and number of such spines differ across echinostome species. This can also contribute to variation in attachment strength among species independently of spine numbers. Our analysis also revealed an effect of the definitive host taxon used by echinostomes and an interaction between body size and host taxon used. In particular, the positive effect of body size is stronger for echinostomes using ectotherms as definitive host. It is possible that attachment strength needs to scale more strongly with body size for worms living in ectothermic hosts because of the nature of their gut lining, or because of the physical properties of the digested food passing along the gut. Regardless of these differences among echinostomes using different taxa of definitive hosts, our study supports the presumed role of collar spines as anchoring structures (Fried et al., 2009), and provides evidence that their number has been in part driven by natural selection to match the attachment strength required for the worm’s body size. This finding has parallels with the positive interspecific correlation among acanthocephalan species between worm body size and the number of hooks on their proboscis (Poulin, 2007), the positive interspecific correlation among species of the cestode genus *Acanthobothrium* between worm body size and the total size of attachment structures (Randhawa and Poulin, 2010), and the strong intraspecific correlation between worm body size and the number of attachment clamps per worm in the ectoparasitic monogenean *Sparicotyle chrysophrii* (Villar-Torres et al., 2023). The latter example reflects developmental adjustments whereas the former 2, like our result, reflect evolutionary adaptation; however, all provide evidence that the number of attachment structures in parasitic helminths matches the worms’ functional requirements.

Finally, we tested whether phylogenetic conservatism may explain variation in the number of collar spines among echinostomes (Pagel, 1999). If this trait is phylogenetically conserved, we would expect closely related species and genera to have similar numbers of collar spines due to shared evolutionary history and inheritance from a common ancestor. We indeed found evidence of strong phylogenetic conservatism in spine numbers. This reinforces the use of this trait by taxonomists for the tentative placement of new species into a particular genus (Kostadinova and Gibson, 2000; Kostadinova, 2005). Based on a look at the dataset we compiled, it is noticeable that at the lower end of the spectrum of spine numbers (i.e. taxa with 23–27 spines), there is much consistency, however those genera with higher numbers of spines seem to be much more variable both intra- and interspecifically. Furthermore, we found that branches leading to species with substantially higher numbers of spines are nested within clades with low spines numbers or vice versa. This suggests that divergence from the ancestral trait value does occur. Based on the relationship with body size we observed, evolutionary changes in spine numbers may be linked with changes in body size and the associated changes in required attachment strength.

In summary, we first confirmed that the arrangement of spines (2 identical arcs separated by a central gap vs an uninterrupted collar) determines whether a species possesses an even or odd spine number; exceptions are likely to be the product of missing, retracted or supernumerary spines in the specimens examined. The excess of prime numbers among species with an odd number of spines is best explained by chance. Second, we showed that spine numbers covary positively with body size among species, suggesting the action of natural selection and a functional adaptive role for the spines in attachment of the worms to the host’s gut wall. Finally, we also confirmed that spine number is a phylogenetically conserved trait, with related species sharing very similar spine numbers, although with some exceptions. Overall, it seems like the number of collar spines, a distinguishing characteristic of echinostome trematodes, is a compromise between the conservative force of phylogenetic inheritance over evolutionary history and the driving force of natural selection in response to changes in worm sizes.

## Supporting information

Presswell et al. supplementary material 1 Presswell et al. supplementary material

Presswell et al. supplementary material 2 Presswell et al. supplementary material

## Supplementary material

The supplementary material for this article can be found at https://doi.org/10.1017/S0031182025000046.
