# Supplementary material for: Size, spines, and primes: the drivers of collar spine numbers among echinostome trematodes
Source: Parasitology. 2025 Jan 27;152(1):123–31. doi: 10.1017/S0031182025000046 (PMC12088915; doi:10.1017/S0031182025000046)
Supplement: Presswell et al. supplementary material 2 — Presswell et al. supplementary material [file S0031182025000046sup002.docx]

SUPPLEMENTARY MATERIAL

**Size, spines, and primes: the drivers of collar spine numbers among echinostome trematodes**

Bronwen Presswell, Priscila M. Salloum, Jerusha Bennett, Katherine E. Buschang, and Robert Poulin

Table S1. Likelihood ratio test comparing the two main effects generalized linear models (GLMs) using either a Poisson or negative binomial structure. DF: degrees of freedom; LogLik: log-likelihood; Likelihood ratio: computed based on the chi-square statistic.

| Model | DF | LogLik | Difference in DF | Likelihood ratio | Pr(>Chisq) | Significance |
| --- | --- | --- | --- | --- | --- | --- |
| Poisson | 4 | -2191.5 |  |  |  |  |
| Negative Binomial | 5 | -1952.1 | 1 | 478.86 | < 2.2e-16 | *** |

Table S2. Distribution of deviance residuals, AIC, null deviance and residuals deviance when comparing the main effects model (spine number ~ body surface area + host taxa) with the interaction model (spine number ~ body surface area * host taxa). Min: Minimum residuals deviance; 1Q: first quartile of residuals deviance; Median: median of residuals deviance; 3Q: third quartile of residuals deviance; Max: maximum residuals deviance; AIC: Akaike Information Criterion; DF: degrees of freedom.

| Model | Min | 1Q | Median | 3Q | Max | AIC | Null deviance  [DF] | Residuals deviance  [DF] |
| --- | --- | --- | --- | --- | --- | --- | --- | --- |
| Main effects | -5.1518 | -1.4439 | -0.4747 | 0.9831 | 7.3276 | 4391 | 1649.2  [534] | 1561.1  [531] |
| Interaction | -4.9657 | -1.4322 | -0.4733 | 0.9642 | 7.3233 | 4376.1 | 1649.2  [534] | 1542.2  [529] |

Table S3. Likelihood ratio test comparing the main effects and the interaction generalized linear models (GLMs). DF: Degrees of freedom of each model; LogLik: log-likelihood; Likelihood ratio: computed based on the chi-square statistic.

| Model | DF | LogLik | Difference in DF | Likelihood ratio | P-value | Significance |
| --- | --- | --- | --- | --- | --- | --- |
| Main effects | 4 | -2191.5 |  |  |  |  |
| Interaction | 6 | -2182.1 | 2 | 18.896 | 7.883e-05 | *** |

Table S4. Species of Superfamily Echinostomatoidea and GenBank Accession numbers used in the 28S phylogenetic analysis.

| Species name | GenBank Accession |
| --- | --- |
| **Family: Caballerotrematidae** |  |
| *Caballerotrema brasiliense* | PQ114584 |
| **Family: Echinochasmidae** |  |
| *Echinochasmus beleocephalus* | KT956929 |
| *Echinochasmus coaxatus* | KT956928 |
| *Echinochasmus donaldsoni* | KT956930 |
| *Echinochasmus japonicus* | JQ890579 |
| *Echinochasmus milvi* | KT873319 |
| *Echinochasmus mordax* | KT956931 |
| *Echinochasmus perfoliatus* | OR532445 |
| *Echinochasmus pseudobeleocephalus* | OR076694 |
| *Echinochasmus suifunensis* | MT447057 |
| *Stephanoprora amurensis* | MT447053 |
| *Stephanoprora aylacostoma* | PQ137075 |
| *Stephanoprora chasanensis* | KT873321 |
| *Stephanoprora pseudoechinata* | KT956934 |
| *Stephanoprora uruguayensis* | PQ137070 |
| *Uroproctepisthmium bursicola* | KT956938 |
| **Family: Echinostomatidae** |  |
| *Artyfechinostomum malayanum* | OR509026 |
| *Artyfechinostomum sufrartyfex* | KF781303 |
| *Chaunocephalus ferox* | KT447522 |
| *Drepanocephalus mexicanus* | MF351543 |
| *Drepanocephalus spathans* | MF351546 |
| *Echinoparyphium aconiatum* | KT956912 |
| *Echinoparyphium cinctum* | AF184260 |
| *Echinoparyphium ellisi* | KY436410 |
| *Echinoparyphium mordwilkoi* | KJ542642 |
| *Echinoparyphium poulini* | KY436409 |
| *Echinoparyphium recurvatum* | KT956913 |
| *Echinostoma bolschewense* | KP065592 |
| *Echinostoma caproni* | AF026104 |
| *Echinostoma chankense* | MT577829 |
| *Echinostoma cinetorchis* | KX817348 |
| *Echinostoma maldonadoi* | OQ132569 |
| *Echinostoma miyagawai* | KT956916 |
| *Echinostoma nasincovae* | KP065603 |
| *Echinostoma novaezealandense* | KY436407 |
| *Echinostoma paraensei* | EU025867 |
| *Echinostoma paraulum* | KP065604 |
| *Echinostoma pseudorobustum* | OK586835 |
| *Echinostoma revolutum* | KP065596 |
| *Echinostoma trivolvis* | AY222246 |
| *Euparyphium albuferensis* | AY219697 |
| *Euparyphium capitaneum* | KP009620 |
| *Euparyphium murinum* | KT956917 |
| *Hypoderaeum conoideum* | KT956918 |
| *Isthmiophora hortensis* | AB189982 |
| *Isthmiophora melis* | AF151941 |
| *Moliniella anceps* | KT956921 |
| *Neoacanthoparyphium echinatoides* | KT956922 |
| *Neomoliniella longicorpa* | OP410309 |
| *Neopetasiger islandicus* | KT831344 |
| *Neopetasiger neocomense* | OL470525 |
| *Patagifer bilobus* | KT956945 |
| *Patagifer vioscai* | KT956946 |
| *Pegosomum asperum* | KY945919 |
| *Pegosomum saginatum* | KY945918 |
| *Petasiger exaeretus* | KT956923 |
| *Petasiger phalacrocoracis* | KT956926 |
| *Petasiger radiatus* | KY284010 |
| *Prionosomoides phrynopsis* | OR135717 |
| **Family: Himasthlidae** |  |
| *Acanthoparyphium shinanense* | MZ146319 |
| *Acanthoparyphium spinulosum* | KT956939 |
| *Himasthla continua* | MT987601 |
| *Himasthla leptosoma* | KT956942 |
| *Himasthla limnodromi* | KT956943 |
| *Himasthla militaris* | KT956944 |
